# Indigenous obesity in the news: a media analysis of news representation of obesity in Australia’s Indigenous population

**DOI:** 10.1186/s40608-016-0109-1

**Published:** 2016-06-23

**Authors:** Salwa Islam, Lisa Fitzgerald

**Affiliations:** School of Public Health, University of Queensland, Herston Road, Herston, Qld 4006 Australia

**Keywords:** Media analysis, Indigenous, Obesity, Representation, Framing

## Abstract

**Background:**

High rates of obesity are a significant issue amongst Indigenous populations in many countries around the world. Media framing of issues can play a critical role in shaping public opinion and government policy. A broad range of media analyses have been conducted on various aspects of obesity, however media representation of Indigenous obesity remains unexplored. In this study we investigate how obesity in Australia’s Indigenous population is represented in newsprint media coverage.

**Method:**

Media articles published between 2007 and 2014 were analysed for the distribution and extent of coverage over time and across Indigenous and mainstream media sources using quantitative content analysis. Representation of the causes and solutions of Indigenous obesity and framing in text and image content was examined using qualitative framing analysis.

**Results:**

Media coverage of Indigenous obesity was very limited with no clear trends in reporting over time or across sources. The single Indigenous media source was the second largest contributor to the media discourse of this issue. Structural causes/origins were most often cited and individual solutions were comparatively overrepresented. A range of frames were employed across the media sources. All images reinforced textual framing except for one article where the image depicted individual factors whereas the text referred to structural determinants.

**Conclusion:**

This study provides a starting point for an important area of research that needs further investigation. The findings highlight the importance of alternative news media outlets, such as The Koori Mail, and that these should be developed to enhance the quality and diversity of media coverage. Media organisations can actively contribute to improving Indigenous health through raising awareness, evidence-based balanced reporting, and development of closer ties with Indigenous health workers.

**Electronic supplementary material:**

The online version of this article (doi:10.1186/s40608-016-0109-1) contains supplementary material, which is available to authorized users.

## Background

Obesity is a significant issue for Indigenous populations around the world [1], with many countries reporting higher obesity rates amongst Indigenous populations than overall population rates [2–5]. Obesity is a major contributor to Indigenous morbidity and mortality in Australia [6–8], and is a significant risk factor for a range of illnesses such as Type II diabetes, osteoarthritis, cardiovascular disease and some cancers [9]. Excessive weight was the second highest contributor to the total burden of disease and injury amongst Indigenous Australians in 2003 [9, 10], and obesity is a major contributor to the lower life expectancy experienced by Indigenous Australians [11].

In news media coverage and public discourse, obesity is often purported to be a ‘lifestyle’ issue [12, 13], however there are a number of structural determinants such as social, economic, political, environmental and technological factors that influence an individual’s likelihood of becoming obese [9]. Disadvantaged socioeconomic status (SES), a lack of post-school qualifications, ethnicity and geographic location are some of the structural determinants of obesity [9]. Indigenous obesity is influenced by a range of external factors including colonisation, low SES, and difficulties in accessing healthy food [7, 9, 14], some of which are unique to this population. Indigenous heritage is itself a risk factor of obesity [7, 9, 14] and the greater burden of disease experienced by this population exists throughout the lifecycle [7]. This is further exacerbated by the presence of other structural determinants such as low SES [7, 9], a group in which Indigenous Australians are disproportionately represented [7]. Accessing nutritious foods can be challenging for Indigenous Australians in low income groups and/or living in rural and remote locations [7, 9, 14], or where government regulations limit access to traditional foods [7]. The effects of colonisation is a determinant of health uniquely faced by Indigenous populations worldwide [7, 14], with Indigenous Australians experiencing a dramatic change in lifestyle after European settlement and ongoing adverse impacts on health and wellbeing [7].

The media can play a significant role in shaping public opinion and even government policy [15–21], using language devices and visual imagery to represent issues in a way that subtly supports a particular perspective [22–25]. This is especially relevant to obesity where excessive media focus on individual responsibility or causes and solutions can reinforce weight stigma, and distract attention away from structural determinants, governmental and societal responsibility, and societal-level solutions [19, 20]. The representation of obesity in the media is a topical issue in Australia and many countries around the world [15, 19, 20, 23, 26–29]. In a large-scale study in the United Kingdom, Hilton et al. [27] observed increasing coverage of the ‘obesity epidemic’ and a shift from individualistic frames to more structural frames, perhaps indicating an inclination towards regulatory change in the public discourse. Gollust et al. [26] found an increase in representation of non-white individuals and a decrease in depictions of stereotypical behaviours over time in media images of obesity printed alongside articles in US news magazines. The authors also noted that an underrepresentation of Latinos, African Americans and elderly obese still remained, and that a variety of reasons ranging from pushing policy change to journalistic values could be responsible for the patterns observed [26]. McClure et al. [20] and Puhl et al. [19] also investigated media representation of obesity in US news coverage, specifically focussing on images [20] and video content [19], respectively. In contrast to Gollust et al.’s [26] findings, both Puhl et al.’s [19] and McClure et al.’s [20] studies observed stigmatising imagery of obesity, raising concerns regarding negative public opinion and general treatment of obese individuals, internalising of negative stereotypes by obese individuals, and the resulting physical and psychological harm. De Brun et al.’s [30] study observed increasing coverage of obesity in an Irish newspaper, which was predominantly framed as a ‘lifestyle’ issue although structural frames did increase during the study period. Similarly, Lawrence [31] observed a shift in news media representation of obesity from that of individual causes and personal responsibility, to one of environmental causes. However, Lawrence [31] also noted that the increasing citation of environmental causes was being met with an increase in personal responsibility frames in response. In contrast, Holmes [28] observed an unusual trend in Canada where the ‘obesity epidemic’ was not framed as an individual problem but rather as a collective challenge for the nation to overcome. Obesity was found to be framed as an individual ‘lifestyle’ issue in Australian media [12, 15]; and in the case of childhood obesity, the responsibility was often assigned to parents [12, 15], and one study found it to be solely attributed to mothers [13].

Recent research has identified the significant influences of media framing of Indigenous issues on the Australian political and public spheres [16, 25, 32–36], however McCallum has also noted that literature exploring media representation of Indigenous health is limited [16]. Hollinsworth [34] argues that media framing of Indigenous Australians is particularly influential, as many people have little other involvement with the Indigenous community. There is evidence that Indigenous Australians are portrayed negatively in media representations and the framing of Indigenous issues “is a form of racist discourse” [16, p336]. Racial frames are commonly employed by media sources when reporting on Indigenous people, and can be used to link racial identity with negative, anti-social or criminal behaviours [35]. Negative coverage of Indigenous Australians including regarding health [16, 25] has been found to have significant consequences for Indigenous people [34, 36], for example by influencing government policy [36] and social attitudes [34]. McCallum’s [16, 32] studies of the shift in news media reporting of Indigenous health over time found that government policies changed dramatically in response to shifts in ‘alarmist’ media framing. McCallum [16, 32] provided the Northern Territory Emergency Response (the ‘Intervention’) in 2007 as an example of the profound interconnectedness between media representation and government policy. This example saw the introduction of “radical policy solutions” [16], (p332) involving the engagement of the military to enforce a range of coercive measures in remote Northern Territorian communities [16], (p333), in response to an overemphasised ‘Indigenous health crisis’ frame represented in media reporting [16].

A review of current literature highlights the scarcity of research studying media representations of Indigenous health. Extensive searching failed to identify any papers investigating media and Indigenous obesity, despite obesity being known to significantly impact on Indigenous health and wellbeing [9–11], and strong evidence that both obesity and the influence of media are important issues facing our society today. The following media analysis aims to shed light on the issue of media representations of Indigenous obesity in Australia and contribute to filling this gap in the literature.

Content analysis is a method that uses clearly defined criteria to analyse news media material, providing useful insights into the content and context of news media articles by exploring underlying meanings and framing of content, and the broader implications of the representation of an issue [37]. Framing is a strategy whereby communicators, consciously or subconsciously, select and promote certain facts or points of view, with the aim of increasing the salience of the content to the audience [15, 18, 23, 28, 35]. It is often utilised as a means of defining problems, diagnosing causes, identifying solutions and making moral judgements [18]. Media outlets employ common ideological constructions to provide a context with which to frame news stories in a manner that is easily understood by their audiences [28, 35]. Although audiences are able to choose their own constructs or opinions of issues, framing is particularly influential to audience perceptions of issues where the audience is not as well-informed or active participants of the issue being reported [17, 18, 21]. Image framing is an often overlooked but important component of news media framing that can assist in conveying ideas, eliciting strong emotional responses from audiences, reinforcing stereotypes and guiding audience perceptions and understandings of an issue [26, 29]. This can even be the case where a biased image is accompanying otherwise neutral content [19, 20]. Images can also be used to frame issues in a manner which may be deemed too controversial or may not be expressed in textual form, for example racial, gender or demographic profiling [26]. Image framing can be particularly influential on public opinion as images are often accepted as reflective of reality, and news is reportedly better understood by the public through images and videos rather than written or audio content [19]. The framing of an issue lends itself to subtly pushing particular viewpoints especially when widespread across various media outlets and media (e.g. print, television, online, radio, etc.) which, unless actively countered or highlighted, can influence how the issue plays out in the public discourse [15, 23, 29].

In this paper we utilise content and framing analysis to explore how obesity in Australia’s Indigenous population is represented in both general and Indigenous-focussed news media coverage, and potential implications for the public discourse of Indigenous obesity. The investigative questions include:

- What is the extent of media coverage of this issue?
- How is this coverage distributed over time and across media sources?
- How is Indigenous obesity represented by the media, particularly the causes/origins of and solutions to obesity?
- How is this issue framed in both text and image content?

## Methods

A number of studies have been used to guide the development of the method of this research. Atanasova et al. [29] examined media analyses of obesity representation and provided a number of recommendations to improve the quality and scope of the literature, some of which are incorporated into this study. The authors identified a need for multimodal studies [29] which is addressed in this paper by including 1) framing analysis of both text and images in printed media, and 2) a comparison of coverage by mainstream and Indigenous media sources. The first aspect ensures a more accurate understanding of framing and representations [26, 29], and allows the study to analyse whether images included in news articles reinforce or contradict the textual content [29]. The second aspect provides the opportunity to consider the contributions of mainstream and Indigenous media on the overall media discourse [35]. The use of emotion-eliciting language in articles [29] is also analysed.

### Analysis

A combination of both quantitative content analysis and qualitative framing analysis was utilised to broaden the depth of the study [16, 27]. The quantitative analysis examined aspects such as reporting trends over time [16, 27, 32] and the distribution of articles according to media outlet [23] through frequency distribution analyses. Each news article was assigned an identification number and the database, source (media organisation and outlet), year and date, title, section, and content type were recorded. The qualitative analysis consisted of coding the representations of the causes/origins of and solutions to Indigenous obesity in each media article. Each article was classified as ‘individual/behavioural’, ‘structural/social’, ‘genetic/biological’, according to what the causes/origins and/or solutions were attributed to, or ‘combined’ where multiple frames were used. Articles where no causes or solutions were clearly articulated were classified ‘N/A’. The above categories have been drawn from a number of sources [15, 27–29, 35]. Textual framing analysis was conducted by examining the language, common meanings/understandings, and any devices such as metaphors, hyperbole, repetition, catchphrases, and emotion-eliciting language used to create frames in the articles [15, 17, 23, 28, 29, 35]. Image framing analysis was performed using criteria adapted from Gollust et al [26] and McCallum [16], and included assessment of image features such as weight, sex, race, SES, age and framing of obesity. The framing of obesity was assessed on whether the image was non-stigmatising/generic/neutral (e.g. images without people, inanimate objects) or stigmatising (e.g. emphasising/focussing on individual body parts such as the abdomen or excluding the head, or showing shirtless/ill-fitting clothes), and what activity was portrayed (e.g. eating ‘healthy’/‘unhealthy’ food, sedentary/physical/everyday activities). The construction of stories and dominant views presented was investigated by identifying which voices were included or prominent in the articles (e.g. academics, nutritionists, community leaders, politicians, members of the public, etc.) [16, 28, 38], and how these voices contributed to the frames of the articles [38]. The quantitative, qualitative and framing analyses were combined to identify the overall framing of articles, distribution of frames according to media source, and which frames or sources dominated the discourse. The image and textual framing were compared to identify whether the images supported or contradicted the textual frames.

### Sampling

Relevant articles were located using the *Factiva* and *Infokoori* databases. *Factiva* provides access to a wide range of newsprint articles, and the search was limited to articles from the Australian region, in English, that included the search terms in the headline and lead paragraph. *Infokoori* includes an index to the *Koori Mail*, a fortnightly national Indigenous newspaper and time was the only search limit applied. The searches were conducted using a seven year timeframe from 2007–2014 to ensure a large enough, but manageable sample. After testing for sensitivity, four searches were conducted using the Boolean search method in *Factiva* with the following search terms^1^:

1. “indigenous AND obes*”^2^
2. “indigenous AND fat”
3. “aboriginal AND obes*”
4. “aboriginal AND fat”

**Fig. 1:**
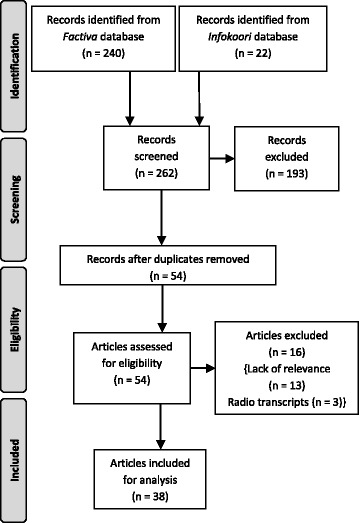
Sampling flowchart. Chart template adapted from www.prisma-statement.org

*Infokoori* was searched with the terms “obesity” and “fat”. The sampling process is outlined below in Fig. 1,^3^:

A total of 262 articles were screened, and a final sample of 38 articles was eligible for analysis. Of the 69 articles included after preliminary screening, 15 articles were duplicates and a further 16 articles were excluded during eligibility assessment as the articles were radio transcripts or lacked relevance. The final sample included for analysis consisted of 9 *Infokoori* articles and 29 *Factiva* articles. Media websites and microfiche holdings were used to locate as many original articles as possible, including images. Original articles were obtained for a total of 22 articles, with 8 articles including images. As coding involves a degree of subjectivity [26], inter-rater reliability of the coding was assessed by selecting a sample of 11 articles (29 % of the total sample) which were then independently coded by authors SI and LF, LF being an experienced media analysis researcher. Coding for the articles sampled was then compared and 100 % agreement was observed. The sample was also cross-checked by both authors to ensure the quality and accuracy of the data.

## Results

**Fig. 2:**
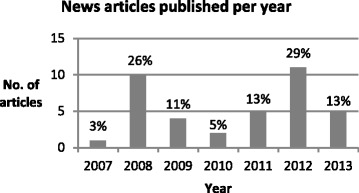
Proportion of total articles published each year, 2007–2013

**Fig. 3:**
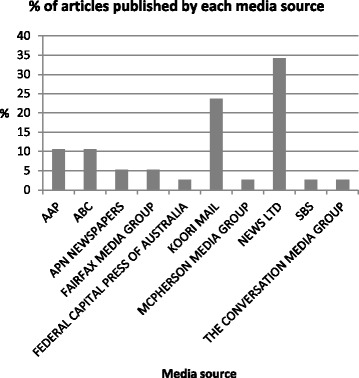
Proportion of total articles published by each media source

The number of articles published varied considerably over the years (Fig. 2), with 2008 and 2012 responsible for more than half the total articles (55 %). In some years only one or two articles were published throughout the whole year and large gaps of many months were observed, with one gap spanning 14 months. The ratio of articles published by the Indigenous media source and mainstream sources was approximately 1:3 (24 % and 76 %, respectively). This is surprising as the Indigenous media source, *Koori Mail*, is published fortnightly compared with the broad range of mainstream sources, many of which are published daily. *News Ltd* had by far the most coverage publishing 34 % of the articles (Fig. 3); however these were distributed across a number of outlets (13 articles across 9 outlets).

**Fig. 4:**
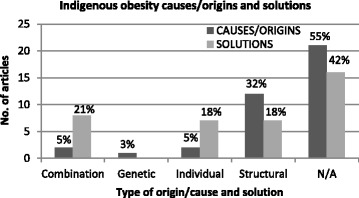
Distribution of articles according to causes/origins and solutions represented

Structural causes/origins were most often referred to in the articles where causes were given (12 articles, 32 %), and genetic causes/origins were least cited (1 article, 3 %) (Fig. 4). The majority of articles (21 articles, 55 %) did not provide a cause/origin at all, however causes/origins were alluded to though not explicitly stated in some articles and therefore were included in the ‘N/A’ category also. A range of structural causes/origins were mentioned including food insecurity/poverty, disadvantaged/remote location, and a lack of access to healthy food/fresh produce and health services. Unhealthy diet and sedentary lifestyle/inactivity were the most common individual factors identified. The genetic tendency towards abdominal fat distribution amongst Indigenous people, especially those with longer-limbed structures, was the only genetic cause/origin cited. Some articles made reference to factors such as decline in traditional lifestyles and higher rates of certain health conditions amongst Indigenous Australians. Further details of the causes/origins cited in the articles and distribution across media sources are included as additional materials.

Many news articles did not clearly cite any solutions to Indigenous obesity (42 % of articles), although solutions may have been alluded to. News articles referring to structural solutions, individual solutions, or a combination of the two were almost equally split (18 %, 18 %, and 21 % respectively) meaning that the solutions were much more evenly distributed across the categories than the causes/origins. Given that structural causes/origins were most frequently cited (12 out of 17, 71 %), individual solutions appear to be over-represented and/or structural solutions under-represented. The individual solutions represented in the articles included healthy diet, exercise/physical activity, losing weight and lap-band surgery. The structural solutions included working together with communities (multi-sectoral approach), improving accessibility to fresh produce, financial incentives, and sport/recreation programs. However, even articles where structural solutions (e.g. community programs) were provided, often the focus of these programs were on individual ‘lifestyle’ changes. Further details of the solutions cited in the articles and distribution across media sources are included as additional materials.

A variety of frames were identified in the sample of articles, as outlined below (Table 1). A limited range of voices were observed in the articles, the majority of which were program/survey coordinators, spokespersons or health professionals (24 of 38 articles, 63 %). Other voices featured were those of government ministers/spokespeople and other authorities such as police (7 articles, 18 %), and program participants or members of the public (5 articles, 13 %). Five articles (13 %) did not feature any direct voices and the remaining 4 articles (11 %) featured the views of the author.

**Table 1:** Media framing of obesity

Frame	Description	Articles *n* (%)
Structural determinants	• Obesogenic environments, external/extrinsic factors and/or structural determinants represented as causes/solutions of obesity • Indigenous Australians identified as *not* lacking motivation to be healthy; framed as facing many hurdles/challenges beyond their control preventing them from being healthy	17 (45)
‘Good news’ stories	• Positive stories reporting on donations, or successful programs, trials or studies • Promoting efforts of governments (state, local) or other organisations	15 (40)
‘Lifestyle’ issue	• ‘Lifestyle’ identified or alluded to as the solution to Indigenous obesity • Most common suggestions were dietary changes and increasing physical activity/exercise	12 (32)
Risk factor of other diseases	• Obesity identified as a risk factor or cause of other diseases (e.g. diabetes, cancer, kidney disease) • Some articles used emotive, sensationalising or alarmist language, and/or statistics to emphasise frame	12 (32)
Willpower and determination	• Obesity framed as an individual issue and a matter of choice • Willpower presented as the only prerequisite to overcome obesity; and that it is lacking • Frame conversely applied to laud individuals/communities for successfully ‘taking control’; framed as possessing such a strong desire to resolve obesity that they defied the odds and succeeded	7 (18)
Statistics	• Statistics used to bolster message of article and frequently highlighted with language features; often described as ‘alarming’ • Indigenous obesity represented as an important issue, however the small number of articles published did not reflect this importance	5 (13)
Working together	• Focus on multi-sectoral approaches to addressing Indigenous obesity • Highlighted governments, organisations, or study/trial coordinators working with local communities; or individuals within communities working together	5 (13)
Back to basics	• Focus on simple, commonsense ‘lifestyle’ changes, food and nutrition, preparing healthy meals, and increasing physical activity • Included encouragement to return to traditional roots and lifestyle	4 (11)
Benefits of weight loss	• Positive results of weight loss or ‘lifestyle’ change highlighted • Success stories featuring emotive language to emotionally draw-in reader and emphasise benefits (e.g. social, psychological, etc.) experienced by individuals	4 (11)
Surgery solution	• Lap-band surgery represented as the solution to obesity; identified as a simple yet effective solution with very positive results • Also included a contested representation where both benefits and risks/concerns were raised	3 (8)
The saviour	• Individuals represented as rescuing Indigenous communities from obesity through financial or other support; portrayed as significantly contributing to addressing Indigenous obesity	2 (5)
Race	• Included both positive and negative representations of the racial frame; race identified as both a source of pride and the cause of ill-treatment	2 (5)

Five news articles with images were suitable for analysis (Table 2). Only one article featured image framing that contradicted the textual frames, where structural determinants were referred to in the text but individual factors were depicted in the image.

**Table 2:** Analysis of imagery and textual framing of Indigenous obesity

Source	Title	Image	Do the image frames support or contradict the textual frames?
*Koori Mail,* 2 December 2009	Defying the trend	Large, colour photograph of WA Health Minister	Supports—image draws attention to the government's contribution to the successful program, employs ‘good news story’, ‘working together’, and ‘willpower or determination’ frames. Image ties in with article title and caption “West Australian Health Minister Kim Hames: Contrary to the Australia-wide trend of rising obesity and diabetes, rates at Looma are not increasing”.
*Courier Mail,* 21 June 2008	‘Heavy' lady wants apology for ‘fat’ note	Very large, black & white photograph of the person concerned (Rube Nixon)	Supports—focus is on Rube’s displeasure over the incident; that she is fighting back and standing up to the mistreatment. Article reinforces ‘race’ frame, voicing her side of the story by focussing on her (image) and her view (text). This is consolidated by the caption focussing on her displeasure (“Took offence… Rube Nixon says police deliberately insulted her”) and title focussing on redress—what Rube is demanding.
*ABC,* 11 October 2008	Taskforce ‘will cut Indigenous health gap’	Small, colour photograph of an overweight Indigenous man (mid-section), holding a cigarette and can of alcohol	Contradicts—article text focuses on ‘structural determinants’ and ‘working with the community’ frames, but a stigmatising image focussing on the mid-section of an overweight/obese Indigenous person, smoking and drinking frames it as an individual ‘lifestyle’ issue. The caption “Closing the gap: tackling tobacco, alcohol-related diseases” supports the image’s ‘lifestyle’ focus whereas the title supports the structural focus of the text.
*The Conversation,* 2 July 2012	Innovative strategies needed to address Indigenous obesity	Two medium-sized, colour photographs: 1) an Australian outback landscape, 2) an Indigenous artwork depicting traditional foods	Supports—the outback landscape and associated caption “Just beyond the built community lies a health-promoting environment providing cultural, spiritual and physical nourishment” point to structural/environmental factors as the solution, highlighting ‘structural factors’ frame. The indigenous artwork and caption “When Indigenous Australians lived a traditional lifestyle, their diets were rich in lean animal foods that provided abundant protein, and sources of slowly digested carbohydrate” supports ‘back to basics’ frame or return to traditional diet as the solution. In both cases the captions, rather than images, and title highlight the theme of ‘innovative’ structural solutions that incorporate traditional Indigenous culture.
*SBS,* 15 December 2012	Food vans promote bush tucker meals	Large, coloured photograph of the chef and another man in the healthy food van	Supports—close-up shot of smiling “celebrity chef” and caption “Indigenous celebrity chef Mark Olive has launched a healthy food van initiative that he hopes will improving (sic) the health of Indigenous Australians” highlights ‘saviour’ frame; focus on cooking touches on ‘back to basics’ frame; broad smiles and energy depicted in the image reinforces ‘good news story’ frame. The title promoting traditional foods and the successful Indigenous chef emphasise Indigenous pride and success of the program.

## Discussion

The media coverage of Indigenous obesity observed in this study greatly differs from coverage observed in media analyses of obesity, where comparatively larger volumes of articles were published [12, 26, 27, 30]. The small number of articles covering Indigenous obesity published during this study’s timeframe concurs with McCallum’s [16] observation that Indigenous health has limited media coverage compared with other health or Indigenous issues. Indigenous obesity is considered a significant problem in academic literature [6–9], and was represented as such within the media articles; however the limited coverage of this issue (38 articles published in 7 years) demonstrates its lack of importance in the media discourse. This finding is supported by McCombs & Shaw’s [21] agenda-setting theory, which details how greater or lesser media coverage of issues has a direct impact on how importantly the issue is perceived by the public. No visible trend in reporting on Indigenous obesity was evident over the study period, nor was there any obvious trend between media sources. These findings contrast with other studies that observed a clear rising trend in media coverage of obesity over time (e.g. see Hilton et al. [27], Lawrence [31]), or distinct representations of Indigenous health between different media sources [16]. However, the apparent lack of trends observed in this analysis may be partly due to the small sample of articles.

An interesting finding of this study was the significant contribution of the Indigenous media source and self-proclaimed “Voice of Indigenous Australia”, the *Koori Mail*. The observation by Budarick and King [35] that niche media sources are underestimated is pertinent to this analysis. In this analysis, the *Koori Mail* made up almost one-quarter of the total number of articles published during the study period—the second largest contributing media source despite only publishing fortnightly—and included the broadest representation of the causes/origins of and solutions to Indigenous obesity. One measure of the quality of news reporting is the range of interpretive frames included in the coverage of an issue [23], so according to this gauge the *Koori Mail’s* coverage of this issue reflects well. Although this publication has a relatively small readership (100,000+), the quantity and nature of coverage of this issue is important as the *Koori Mail* is a publication that has wide readership amongst Indigenous organisations and those working with Indigenous communities, and the significant contribution of this newspaper to the media discourse highlights its potential influence on the overall representation of this issue.

The representations of Indigenous obesity observed in this analysis were similar to those of other studies in many respects; however there were a few points of difference. Structural determinants featured prominently in the representations of the causes/origins of Indigenous obesity in this analysis, which was surprising as a number of media analyses found a strong focus on individual causes/origins and solutions in media representations of obesity [12, 15, 23, 27, 29]. However, individual solutions appeared to be overemphasised in this analysis, as also found by others [12, 27]. A limited number of news articles in this study referred to the benefits of traditional Indigenous diet and lifestyle, in contrast with the academic literature where this aspect was discussed in detail [7, 14]. Obesity as a risk factor for other diseases or as a key contributor to the life expectancy gap between Indigenous and non-Indigenous Australians was a common theme observed in this analysis. This focus concurs with findings in the literature where obesity was reported as a major contributor to the Indigenous life expectancy gap [11], Indigenous morbidity and mortality [6–8], and a significant risk factor for a number of other diseases [9]. Hilton et al. [27] also found that obesity was represented as a risk factor of other illnesses in their media analysis.

In this study, Indigenous obesity was largely represented as being structural in cause/origin indicating media pressure on structural agencies such as governments, industries and other organisations to provide the solutions for this issue. In comparison, individual solutions were overrepresented thus challenging the above view; instead pointing to a media view that ultimate responsibility lies with the individual. This finding is similar to that of Lawrence’s [31] where an increase in environmental causality frames resulted in increasing personal responsibility frames, thus pushing responsibility and pressure from governments and other structural agencies back to the individual. The impact of such a representation on public opinion—if accepted as presented—would be one of blame shifting onto individuals thus drawing attention away from underlying structural issues [12, 15, 27]. Although the audience is free to accept or reject the representation of an issue, past research has found that where the dominant frame remains largely unchallenged, it often becomes the general public view [17]. As such, blaming of individuals and focussing on individual choices can exacerbate the situation through flow-on effects of discrimination and stigmatisation [15, 19, 20].

Some similarities exist in the obesity frames identified in this media analysis and that of others; however the framing observed in this analysis appeared to be more subtle. The framing of lap-band surgery as a quick and easy medical solution identified by Bonfiglioli et al. [12], and the ‘statistics’ and ‘individual’ frames identified by Holland et al. [23] in their analyses, were also observed to varying degrees in this study. A ‘back to basics’ frame observed by Holmes [28] in Canadian media reporting, was similarly observed in this study also. With respect to image framing, the images analysed in this study played an important role in reinforcing the textual messages in all except one article, where the image conveyed a tacit message regarding ‘lifestyle’ choices and, by only featuring the midsection of the individual, conveyed a depersonalised, stigmatising representation of obesity [19, 37]; despite the textual content referring to structural factors. Bastian [15] observed that the mass media is frequently a channel for influential voices to be expressed and that disadvantaged voices are often not heard. This study observed a similar pattern to Bastian [15] where voices from the grassroots level were only occasionally featured and the voices of experts or authorities were most frequently included, highlighting the contribution of influential voices in setting the tone of media discourses.

### Limitations

The most significant limitation of this study was the restricted depth of analysis due to the study’s scope and size constraints. Initially, the study included TV broadcast news stories however during analysis it became apparent that the TV broadcast articles would be overwhelming for the size of this project, and could easily constitute the sole focus of a media analysis. Another limitation of this study was the development of the coding framework, which could be more rigorous like some studies where pilot studies were initially conducted [26, 27, 30].

## Conclusions

This analysis explored the representation of Indigenous obesity in Australian media coverage and the findings raise a number of interesting points. Firstly, the review of academic literature investigating media representations of Indigenous obesity did not produce any relevant results, suggesting that this may be the first such media analysis and may provide a good starting point for this area of research. There is arguably a need for further research into this issue and a variety of aspects could be investigated, for example comparing media coverage of Indigenous obesity with that of Indigenous health or obesity coverage, and examining the representation of this issue in other media channels (e.g. TV broadcast, radio, or social media). Secondly, this study highlights the importance of active encouragement and development of alternative media organisations to enhance the quality and diversity of the public discourse. Thirdly, media organisations can, and should play an active role in Indigenous health, by raising awareness of Indigenous health issues and objective reporting of areas of need and progress. Fourthly, the most common voices featured in the news media coverage of Indigenous obesity were those of Indigenous health workers and program/study co-ordinators and spokespeople, therefore it is essential that these individuals capitalise on this coverage by raising awareness and presenting evidence-based, balanced views of the issue. There is also a need for media organisations to engage with the wider community by including more voices from the grassroots level. Finally, the development of closer ties between Indigenous health workers and media organisations should be encouraged as the media can play a critical role in Indigenous health, and the two parties should be united by the common goal of advancing Indigenous health. There is compelling evidence that obesity is an important issue affecting the health of Indigenous populations around the world, and that media representation of issues can have significant implications for society. It is hoped that this issue will become an important field of both obesity and media research, and a source of enhancing our understanding of the drivers of Indigenous obesity and the contribution of media to this issue.

## Abbreviations

SES, socioeconomic status, N/A, not applicable

## Additional file

Additional file 1: ADDITIONAL MATERIALS – detailed data tables. (DOCX 29.3 kb)

## Footnotes

1 Search 3 and 4 only included the word Aboriginal and not Aboriginal and Torres Strait Islander as using both terms dramatically reduced the relevance of results, producing a lot of irrelevant results. All articles resulting from a test search of “Torres Strait Island AND obes*” were found to be present in the “aboriginal AND obes*” search, therefore only ‘aboriginal’ was used to conduct the search.

2 ‘*’ was used in searches 1 and 3 to capture all relevant words including obese, obesity, obesogenic, etc.

3 In this figure, n represents the number of studies
