# Supplementary material for: Indigenous obesity in the news: a media analysis of news representation of obesity in Australia’s Indigenous population
Source: BMC Obes. 2016 Jun 23;3:30. doi: 10.1186/s40608-016-0109-1 (PMC4917929; doi:10.1186/s40608-016-0109-1)
Supplement: Additional file 1: — ADDITIONAL MATERIALS – detailed data tables. (DOCX 29.3 kb) [file 40608_2016_109_MOESM1_ESM.docx]

**ADDITIONAL MATERIALS – detailed data tables**

Data Table 1: Origins/causes of Indigenous obesity as represented in each news article

| **SOURCE & DATE** | **TITLE & SECTION** | **ORIGINS/CAUSES** | **EXAMPLE** |
| --- | --- | --- | --- |
| *The Australian* (News Ltd), 23 January 2007 | Tycoon's outback health rescue, *Local* | Structural | - Regional areas lack access to quality health services |
| *Koori Mail* (Independent), 16 January 2008 | When being a loser makes you a winner, *Comment* | Individual | - Over-indulgence and sedentary/unhealthy lifestyle |
| *AAP General News* (AAP), 21 June 2008 | Qld: Outrage after police call woman 'fat and black', *Unclassified* | N/A | - No causes clearly stated |
| *The Courier Mail* (News Ltd), 21 June 2008 | Heavy' lady wants apology for 'fat' note, *News* | N/A | - No causes clearly stated |
| *Koori Mail* (Independent), 27 August 2008 | Addressing obesity the goal of school survey, *Health* | N/A | - No causes clearly stated |
| *Koori Mail* (Independent), 10 September 2008 | Some important food for thought, *Comment* | Combination | - Individual: unhealthy eating and lack of physical activity; lack of time - Genetic: higher risk of certain health conditions amongst Aboriginal people |
| *Newcastle Herald* (Fairfax), 13 September 2008 | Healthy food too costly: professor, *News* | Structural | - Unhealthy foods often cheaper than healthy foods (e.g. fruits/vegetables) - Supermarkets, fast food chains open 24hrs/7days - Access to/cost of health care: dieticians not covered by Medicare so people less likely to get help |
| *ABC News* (ABC), 11 October 2008 | Taskforce 'will cut Indigenous health gap', *Unclassified* | N/A | - No causes clearly stated |
| *ABC News* (ABC), 30 October 2008 | 60pc of adult Aboriginals are overweight: ABS, *Unclassified* | N/A | - No causes clearly stated |
| *Koori Mail* (Independent), 19 November 2008 | Cancer link to weight, *Health* | N/A | - No causes clearly stated |
| *ABC News* (ABC), 8 December 2008 | Obesity inquiry visits far west NSW, *Unclassified* | Structural | - Availability of fresh produce |
| *ABC News* (ABC), 13 October 2009 | Concerns over Indigenous lap band trial, *AM* | Structural | - Food insecurity |
| *AAP General News* (AAP), 16 November 2009 | Healthy food needed on outback shelves: report finds,  *Unclassified* | Combination | - Structural: inadequate access to fresh and healthy food at reasonable cost; Increased reliance on community stores for food supply due to decline in traditional hunting practices - Individual: substantial proportions of incomes spent on sweets, sugared drinks and cigarettes |
| *The Australian* (News Ltd), 30 November 2009 | Children buck health trend, *Local* | N/A | - No causes clearly stated |
| *Koori Mail* (Independent), 2 December 2009 | Defying the trend, *Health* | N/A | - No causes clearly stated |
| *Koori Mail* (Independent), 27 January 2010 | Shedding weight is worth it, *Unclassified* | Individual | - Unhealthy diet, sedentary lifestyles |
| *Southern Courier* (News Ltd), 4 May 2010 | A big fella's film, *Local* | N/A | - No causes clearly stated |
| *AAP General News* (AAP), 29 July 2011 | Report highlights child health issues, *Federal news* | Structural | - More likelihood of obesity in remote, poor and indigenous communities - Access to/cost of health services |
| *The Australian* (News Ltd), 30 July 2011 | Poorest odds for remote children, *Local* | Structural | - More likelihood of obesity in remote, poor and indigenous communities - Access to/cost of health services |
| *The Courier Mail* (News Ltd), 30 July 2011 | Poorer kids' health a national shame, *News* | Structural | - More likelihood of obesity in remote, poor and indigenous communities - Access to/cost of health services |
| *Townsville Bulletin* (News Ltd), 30 July 2011 | national snapshot - City vs remote gap: Report highlights health divide, *National* | Structural | - More likelihood of obesity in remote, poor and indigenous communities - Access to/cost of health services |
| *Northside Chronicle* (News Ltd), 7 September 2011 | Program gains funds, *Unclassified* | N/A | - No causes clearly stated |
| *AAP General News* (AAP), 11 May 2012 | Indigenous obesity could 'widen the gap', *Queensland news* | Structural | - Costly fresh fruit and vegetables in remote communities beyond reach of many, but junk food is still cheap |
| *The Advertiser* (News Ltd), 12 May 2012 | Obesity creates health chasm, *News* | Structural | - Healthy eating difficult as fresh fruit and vegetables far costlier in remote communities than junk food |
| *Weekend Courier* (News Ltd), 8 June 2012 | FREE exercise and fitness programs are available in Rockingham and Kwinana after the neighbouring councils launched Life at the Core last week, *Unclassified* | N/A | - No causes clearly stated |
| *The Conversation* (The Conversation Media Group), 2 July 2012 | Innovative strategies needed to address Indigenous obesity, *Health + Medicine* | Structural | - Poverty - Obesogenic environments: high food prices; few healthy fast food options; limited recreational facilities and safe walking paths; inadequate housing/poor cooking and storage facilities; access to information about healthy food - Food stores mainly stock energy-dense, nutrient-poor, high calorie/fat/sugar/salt containing processed foods; and higher prices for healthy foods (nutrient-rich, fresh produce) - Limited finances/food prices influence consumption patterns |
| *Koori Mail* (Independent), 8 August 2012 | Action urged over obesity, *Health* | N/A | - No causes clearly stated |
| *Northern Territory News/Sunday Territorian* (News Ltd), 18 August 2012 | Indigenous health survey, *Unclassified* | N/A | - No causes clearly stated |
| *St George & Sutherland Shire Leader* (Fairfax Media), 24 August 2012 | Health Survey, *General News* | N/A | - No causes clearly stated |
| *Northern Territory News/Sunday Territorian* (News Ltd), 3 September 2012 | Diabetes on rise, *Unclassified* | N/A | - No causes clearly stated |
| *Centralian Advocate* (News Ltd), 13 November 2012 | Health Study, *Unclassified* | N/A | - No causes clearly stated |
| *Shepparton News* (McPherson Media), 20 November 2012 | Weight shed, health better, *News* | N/A | - No causes clearly stated |
| *SBS World News Headline Stories* (SBS), 15 December 2012 | Food vans promote bush tucker meals, *Unclassified* | N/A | - No causes clearly stated |
| *Coastal Views* (APN Newspapers), 15 March 2013 | Project aids indigenous health, *Unclassified* | N/A | - No causes clearly stated |
| *The Daily Examiner* (Grafton) (APN Newspapers), 15 March 2013 | Taking charge of health issues, *Unclassified* | N/A | - No causes clearly stated |
| *Koori Mail* (Independent), 5 June 2013 | Body fat link to kidney illness, *Health* | Genetic | - Tendency towards central obesity due to skeletal build |
| *Koori Mail* (Independent), 19 June 2013 | Location link to problems, *Health* | Structural | - Location has an effect on obesity rates - Linked to remoteness and areas of disadvantage |
| *The Canberra Times* (Federal Capital Press Of Australia), 25 July 2013 | Sydney Uni to launch $500m obesity centre, *Unclassified* | N/A | - No causes clearly stated |

Data Table 2: Distribution of representations of origins/causes of Indigenous obesity, according to media source

| **MEDIA SOURCE** | **STRUCTURAL** | **INDIVIDUAL** | **GENETIC** | **COMBINATION** | **N/A** | **TOTAL** |
| --- | --- | --- | --- | --- | --- | --- |
| Koori Mail | 1 | 2 | 1 | 1 | 4 | **9** |
| News Ltd | 5 | - | - | - | 8 | **13** |
| Fairfax Media Group | 1 | - | - | - | 1 | **2** |
| ABC | 2 | - | - | - | 2 | **4** |
| The Conversation Media Group | 1 | - | - | - | - | **1** |
| AAP | 2 | - | - | 1 | 1 | **4** |
| Federal Capital Press of Australia (Canberra Times) | - | - | - | - | 1 | **1** |
| APN Newspapers | - | - | - | - | 2 | **2** |
| SBS | - | - | - | - | 1 | **1** |
| McPherson Media Group | - | - | - | - | 1 | **1** |
| **TOTAL** | **12** | **2** | **1** | **2** | **21** | **38** |

Data Table 3: Solutions of Indigenous obesity as represented in each news article

| **SOURCE & DATE** | **TITLE & SECTION** | **SOLUTIONS** | **EXAMPLE** |
| --- | --- | --- | --- |
| *The Australian* (News Ltd), 23 January 2007 | Tycoon's outback health rescue, *Local* | Combination | - Individual: healthy eating and exercise; sustained lifestyle changes - Structural: the health program (as mentioned in article); improve standard of health coverage in rural and remote areas |
| *Koori Mail* (Independent), 16 January 2008 | When being a loser makes you a winner, *Comment* | Individual | - Weight-loss program - Healthier eating, increasing physical activity |
| *AAP General News* (AAP), 21 June 2008 | Qld: Outrage after police call woman 'fat and black', *Unclassified* | N/A | - No solutions clearly stated |
| *The Courier Mail* (News Ltd), 21 June 2008 | Heavy' lady wants apology for 'fat' note, *News* | N/A | - No solutions clearly stated |
| *Koori Mail* (Independent), 27 August 2008 | Addressing obesity the goal of school survey, *Health* | Combination | - Individual: healthy diets - Structural: combined approach of parents and school community encouraging and promoting healthy eating, and boarding schools supplying healthy food |
| *Koori Mail* (Independent), 10 September 2008 | Some important food for thought, *Comment* | Individual | - Healthy diet and exercise - See a nutritionist, or get information from the internet (if accessibility is an issue) |
| *Newcastle Herald* (Fairfax), 13 September 2008 | Healthy food too costly: professor, *News* | Structural | - Cheaper fruits and vegetables so poorer families can afford to eat well - Financial incentives - Involvement of food companies in solution - Food companies and supermarkets providing cheaper healthy food |
| *ABC News* (ABC), 11 October 2008 | Taskforce 'will cut Indigenous health gap', *Unclassified* | Structural | - Shield children from unhealthy food marketing - Work together with Indigenous communities |
| *ABC News* (ABC), 30 October 2008 | 60pc of adult Aboriginals are overweight: ABS, *Unclassified* | N/A | - No solutions clearly stated |
| *Koori Mail* (Independent), 19 November 2008 | Cancer link to weight, *Health* | Individual | - Avoid excessive weight gain - Healthy diet and regular exercise |
| *ABC News* (ABC), 8 December 2008 | Obesity inquiry visits far west NSW, *Unclassified* | N/A | - No solutions clearly stated |
| *ABC News* (ABC), 13 October 2009 | Concerns over Indigenous lap band trial, *AM* | Individual | - Lap-band surgery (although contested in article) |
| *AAP General News* (AAP), 16 November 2009 | Healthy food needed on outback shelves: report finds,  *Unclassified* | Structural | - Provision of quality, reasonably-priced fresh and healthy food by community stores in remote Aboriginal communities - Development of a healthy store policy by stores and local community together - Development of incentive schemes to encourage stores to sell healthy produce - Establishment of an infrastructure fund to assist stores to invest in delivery, refrigeration and storage facilities - Federal government to aid delivery of fresh fruit and vegetables in conjunction with charitable delivery organisations |
| *The Australian* (News Ltd), 30 November 2009 | Children buck health trend, *Local* | Combination | - Structural: the community program (as mentioned in article) including cooking and nutrition classes, wholesome school breakfasts, focus on sport and exercise, and health checks; changes through joint (community) effort - Individual: lifestyle changes |
| *Koori Mail* (Independent), 2 December 2009 | Defying the trend, *Health* | Structural | - Community health program (as mentioned in article) including cooking classes, nutrition programs, school breakfast program, and exercise/sport programs - Communities and government working together |
| *Koori Mail* (Independent), 27 January 2010 | Shedding weight is worth it, *Unclassified* | Individual | - Lose weight, make small lifestyle changes, commit to healthy lifestyle - 10 tips: 1) Eat breakfast, 2) Include vegetables or salad with meals, 3) Choose fruit as a snack, 4) Replace full fat food and drinks with reduced fat alternatives, 5) Choose wholegrain foods in place of more refined ones, 6) Eat smaller serving sizes by using smaller plates and cups, 7) Eat slowly and stop when you are satisfied, not stuffed full, 8) Eat when you genuinely feel hungry rather than for emotional or other reasons, 9) Swap sweetened beverages such as cordial, soft drink and juice with water/diet drinks, 10) Sit at the table to eat your evening meal, not in front of the TV |
| *Southern Courier* (News Ltd), 4 May 2010 | A big fella's film, *Local* | Individual | - Lap-band surgery |
| *AAP General News* (AAP), 29 July 2011 | Report highlights child health issues, *Federal news* | N/A | - No solutions clearly stated |
| *The Australian* (News Ltd), 30 July 2011 | Poorest odds for remote children, *Local* | N/A | - No solutions clearly stated |
| *The Courier Mail* (News Ltd), 30 July 2011 | Poorer kids' health a national shame, *News* | N/A | - No solutions clearly stated |
| *Townsville Bulletin* (News Ltd), 30 July 2011 | national snapshot - City vs remote gap: Report highlights health divide, *National* | N/A | - No solutions clearly stated |
| *Northside Chronicle* (News Ltd), 7 September 2011 | Program gains funds, *Unclassified* | Structural | - Sports and recreation program for Indigenous youths |
| *AAP General News* (AAP), 11 May 2012 | Indigenous obesity could 'widen the gap', *Queensland news* | N/A | - No solutions clearly stated |
| *The Advertiser* (News Ltd), 12 May 2012 | Obesity creates health chasm, *News* | N/A | - No solutions clearly stated |
| *Weekend Courier* (News Ltd), 8 June 2012 | FREE exercise and fitness programs are available in Rockingham and Kwinana after the neighbouring councils launched Life at the Core last week, *Unclassified* | Combination | - Structural: the exercise and fitness program (as mentioned in article) which includes free activities – group exercise (over 50s/outdoor/gym training, walking groups/trails, dance sessions), and lifestyle change programs (HEAL-healthy eating, activity and lifestyle; quit smoking/nutrition education sessions) |
| *The Conversation* (The Conversation Media Group), 2 July 2012 | Innovative strategies needed to address Indigenous obesity, *Health + Medicine* | Structural | - Programs focussing on modifying obesogenic environments - A multi-level, multi-sector integrated approach with government support and resources - Traditional holistic solutions - Economic levers in the form of subsidies rather than taxes - Community working together - Innovative economic strategies |
| *Koori Mail* (Independent), 8 August 2012 | Action urged over obesity, *Health* | Combination | - Structural: collective action against obesity - Individual: healthy choices; regularly visiting the GP; ensuring waist measurements are within recommendations; read food labels; prepare healthy meals; increase physical activity |
| *Northern Territory News/Sunday Territorian* (News Ltd), 18 August 2012 | Indigenous health survey, *Unclassified* | N/A | - No solutions clearly stated |
| *St George & Sutherland Shire Leader* (Fairfax Media), 24 August 2012 | Health Survey, *General News* | N/A | - No solutions clearly stated |
| *Northern Territory News/Sunday Territorian* (News Ltd), 3 September 2012 | Diabetes on rise, *Unclassified* | N/A | - No solutions clearly stated |
| *Centralian Advocate* (News Ltd), 13 November 2012 | Health Study, *Unclassified* | N/A | - No solutions clearly stated |
| *Shepparton News* (McPherson Media), 20 November 2012 | Weight shed, health better, *News* | Individual | - Weight loss via surgical intervention, and education about healthy eating and regular exercise |
| *SBS World News Headline Stories* (SBS), 15 December 2012 | Food vans promote bush tucker meals, *Unclassified* | Combination | - Structural: the health education program (as mentioned in article); educate children about healthy eating; onsite cooking courses - Individual: dietary change; cooking meals ‘from scratch’ |
| *Coastal Views* (APN Newspapers), 15 March 2013 | Project aids indigenous health, *Unclassified* | Combination | - Structural: the community fitness and support program (as mentioned in article) - Individual: weight loss, lifestyle change, healthy eating, physical activity |
| *The Daily Examiner* (Grafton) (APN Newspapers), 15 March 2013 | Taking charge of health issues, *Unclassified* | Combination | - Structural: the community fitness and support program (as mentioned in article) - Individual: weight loss, lifestyle change, healthy eating, physical activity |
| *Koori Mail* (Independent), 5 June 2013 | Body fat link to kidney illness, *Health* | N/A | - No solutions clearly stated |
| *Koori Mail* (Independent), 19 June 2013 | Location link to problems, *Health* | N/A | - No solutions clearly stated |
| *The Canberra Times* (Federal Capital Press Of Australia), 25 July 2013 | Sydney Uni to launch $500m obesity centre, *Unclassified* | Structural | - Clinical, educational or legislative solutions - Empowering individuals through technology |

Data Table 4: Distribution of representations of solutions of Indigenous obesity, according to media source

| **MEDIA SOURCE** | **STRUCTURAL** | **INDIVIDUAL** | **COMBINATION** | **N/A** | **TOTAL** |
| --- | --- | --- | --- | --- | --- |
| Koori Mail | 1 | 4 | 2 | 2 | **9** |
| News Ltd | 1 | 1 | 3 | 8 | **13** |
| Fairfax Media Group | 1 | - | - | 1 | **2** |
| ABC | 1 | 1 | - | 2 | **4** |
| The Conversation Media Group | 1 | - | - | - | **1** |
| AAP | 1 | - | - | 3 | **4** |
| Federal Capital Press of Australia (Canberra Times) | 1 | - | - | - | **1** |
| APN Newspapers | - | - | 2 | - | **2** |
| SBS | - | - | 1 | - | **1** |
| McPherson Media Group | - | 1 | - | - | **1** |
| **TOTAL** | **7** | **7** | **8** | **16** | **38** |
